# Impact of Neointimal Calcifications on Acute Stent Performance during the Treatment of In-Stent Restenosis

**DOI:** 10.5935/abc.20160068

**Published:** 2016-05

**Authors:** Emile Mehanna, Guilherme Ferragut Attizzani, Daisuke Nakamura, Setsu Nishino, Anas Fares, Reem Aoun, Marco Aurelio Costa, Hiram Grando Bezerra

**Affiliations:** Harrington Heart and Vascular Institute, University Hospitals Case Medical Center, Case Western Reserve University - USA

**Keywords:** Optical coherence tomography, in-stent restenosis, neointimal calcification, percutaneous coronary intervention, drug eluting stents

## Abstract

Optical coherence tomography (OCT) has become the invasive imaging modality of choice for coronary stent assessment due to its unmatched spatial resolution. Neointimal calcification (NC) is a rare finding, observed in 5-10% of in-stent restenosis (ISR) neointima. The impact of NC on percutaneous coronary intervention of ISR is unknown. We therefore present the outcome of six unique cases of ISR and NC in which OCT was used to evaluate the impact of NC on the quality of stent-in-stent deployment for the treatment of ISR. This series demonstrates for the first time the impact of NC on stent expansion, a finding which might help guiding percutaneous coronary intervention for ISR with NC.

## Brief Communication

Neoatherosclerosis, defined as the presence of neointimal calcification (NC) or lipid-laden neointima,^1^ has been reported as an important mechanism of late stent failure.^2^ Intravascular imaging modalities enabled further elucidation of neoatherosclerosis´ pathophysiology in vivo.^3^ Neointimal calcification is observed in 5-10% of in-stent restenosis (ISR),^4^ but its impact on the acute performance of stents implanted in-stent for the treatment of ISR is unknown. Intravascular optical coherence tomography (OCT) enables precise assessment of calcified plaques, while dramatically reducing imaging artifacts compared with intravascular ultrasound.^5^ We therefore used OCT to evaluate the impact of NC on the quality of stent-in-stent deployment for the treatment of ISR.

**Figure 1:**
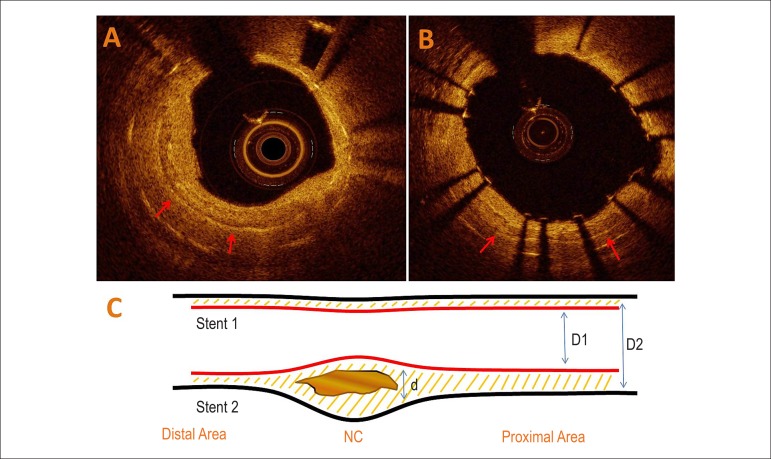
OCT images of in-stent restenosis case with neointimal calcification (red arrow) before (A) and after (B) stent-in-stent implantation. Panel C: schematic representation of the effect of neointimal calcification (NC) on stent expansion. D: stent diameter; d: distance between stents.

Herewith we present 6 cases of ISR and NC from our institution's OCT registry. OCT (C7-XR OCT Intravascular Imaging System; St.Jude Medical, St. Paul, Minnesota) images were acquired pre- and post-stent-in-stent procedure using the integrated automated pullback device at 20 mm/s (frame interval of 0.2 mm). Neointimal calcification was defined as an area of low attenuation, low backscattering and clear borders within the stent neointima (Figure 1). Areas and diameters for the old (outer) and newly implanted (inner) stents were obtained; in addition, the mean distance and area between the stents were automatically obtained by 360° chords (Figure 1). Stent eccentricity was defined as minimum stent diameter/maximum stent diameter, while stent expansion was defined as the average stent area at the NC zone divided by the average stent reference [(average proximal reference + average distal reference)/2]. OCT analysis was undertaken offline by a Core Laboratory blinded to the procedure´s characteristics using commercially available software (Version C.0.4, St Jude Medical, St. Paul, MN). Analyses were concentrated in 3 consecutive frames at 3 different locations (i.e. 9 frames per OCT pullback): 1) NC region, 2) proximal and 3) distal to NC region (Figure 1).

Stent areas, diameters and stent eccentricity were similar between the frames with NC and the frames distal to the NC (Table 1). When comparing the NC area to the ISR region proximal to the NC, there was a trend for smaller area (difference = 0.9 mm^2^, p = 0.09), and diameter (difference = 0.2mm, p = 0.09) of the inner (newly implanted) stent and bigger stent area (difference = 1.2 mm^2^, p = 0.06), and diameter (difference = 0.2mm, p = 0.06) of the external (older) stent at the location of NC compared to the proximal non-calcified ISR analyzed frames (Table 1).

**Table 1:** Quantitative effect of neointimal calcification on stent implantation

	Difference			Difference		
	Proximal – NC	*t*	p-value	Distal – NC	*t*	p-value
Stent 1 Area	0.90	2.11	0.09	0.58	1.52	0.19
Stent 2 Area	-1.20	-2.49	0.06	-0.76	-1.35	0.23
Minimum d	0.03	1.61	0.17	0.02	0.83	0.45
Mean d	0.21	3.90	0.01	0.13	3.21	0.02
Maximum d	0.43	8.30	0.01	0.27	2.06	0.09
Minimum D1	0.17	1.92	0.11	0.09	0.95	0.38
Mean D1	0.20	2.12	0.09	0.12	1.43	0.21
Maximum D1	0.24	2.32	0.07	0.15	1.51	0.19
SE D1	0.02	0.95	0.39	0.02	1.31	0.25
Minimum D2	-0.20	-2.43	0.06	-0.14	-1.31	0.25
Mean D2	-0.20	-2.35	0.06	-0.14	-1.31	0.25
Maximum D2	-0.22	-2.68	0.04	-0.15	-1.45	0.21
SE D2	-0.01	-0.88	0.42	-0.004	-0.38	0.72

Stent 1: inner (newly implanted stent); stent 2: outer (older stent; d: distance between stents; D: diameter of stent; SE: stent eccentricity (minD/MaxD); NC: neointimal calcification.

The mean distance between the stents was always longer at the area of calcification: difference between the NC area and the distal area was 0.13mm (p = 0.02) and the NC area and the proximal ISR region as 0.21 (p = 0.01). The average stent expansion at the area of calcified neointima was 81.4%.

Stent underexpansion has been linked to clinical adverse events, notably stent thrombosis and restenosis.^6,7^ We were able to demonstrate that the presence of NC led to underexpansion of the newly implanted stent compared with adjacent segments. Further investigation is required to determine whether these findings have an impact on clinical events.

The mechanisms leading to stent ISR have been divided into technical (barotrauma outside stented segment, stent gap, residual uncovered atherosclerotic plaques), mechanical (stent underexpansion, non-uniform stent strut distribution, stent fracture, non-uniform drug elution/ deposition, polymer peeling) and biological (drug resistance, hypersensitivity).^8^ The advancement of intravascular imaging, notably OCT, is expected to allow a better understanding of the ISR process and will likely influence the therapeutic strategies (i.e., customized therapy) utilized in this scenario. While current alternatives for ISR therapy (i.e., plain balloon angioplasty, drug-eluting balloon, in-stent DES) are mostly based on the type of restenosis (focal in-stent, focal at stent edge, diffuse in-stent, proliferative),^9^ they do not take neointimal qualitative assessment into account. We believe information provided by OCT imaging could, therefore, further improve therapeutic decisions in ISR. For example, in cases of ISR with NC as herewith described, more aggressive in-stent pre-dilations or use of debulking devices could potentially help improve the expansion of the newly implanted stent. The effect of neointimal atherosclerosis characterization on therapeutic choices for ISR therapy and its effect on clinical outcomes are yet to be determined in future prospective studies.
